# Determination of Metabolomics Profiling in BPA-Induced Impaired Metabolism

**DOI:** 10.3390/pharmaceutics14112496

**Published:** 2022-11-17

**Authors:** Maria Alvi, Kanwal Rehman, Muhammad Sajid Hamid Akash, Azka Yaqoob, Syed Muhammad Shoaib

**Affiliations:** 1 Department of Pharmaceutical Chemistry, Government College University, Faisalabad 38000, Pakistan; 2 Department of Pharmacy, The Women University, Multan 66000, Pakistan; 3 Drugs Testing Laboratory, Faisalabad, Primary & Secondary Healthcare Department, Government of the Punjab, Lahore 54000, Pakistan

**Keywords:** mitochondria, resveratrol, lipid metabolism, amino acid metabolism, gene expression

## Abstract

Exposure to bisphenol A (BPA) is unavoidable and it has far-reaching negative effects on living systems. This study aimed to explore the toxic effects of BPA in an experimental animal model through a metabolomics approach that is useful in measuring small molecule perturbations. Beside this, we also examined the ameliorative effects of resveratrol (RSV) against BPA-induced disturbances in experimental mice. This study was conducted for 28 days, and the results showed that BPA indeed induced an impairment in amino acid metabolism, taking place in the mitochondria by significantly (*p* < 0.05) decreasing the levels of certain amino acids, i.e., taurine, threonine, asparagine, leucine, norleucine, and glutamic acid in the mice plasma. However, the administration of RSV did prove effective against the BPA-induced intoxication and significantly (*p* < 0.05) restored the level of free amino acids. Lipid metabolites, L-carnitine, sphinganine, phytosphingosine, and lysophosphatidylcholine were also determined in the mice serum. A significant (*p* < 0.05) decline in glutathione peroxidase (GPx), superoxide dismutase (SOD,) glutathione, and catalase levels and an elevation in malondialdehyde level in the BPA group confirmed the generation of oxidative stress and lipid peroxidation in experimental mice exposed to BPA. The expression of Carnitine palmitoyltransferase I (CPT-I), carnitine palmitoyltransferase II (CPT-II), lecithin–cholesterol acyltransferase (LCAT), carnitine O-octanoyltransferase (CROT), carnitine-acylcarnitine translocase (CACT), and 5-methyltetrahydrofolate-homocysteine methyltransferase (MTR) genes was significantly upregulated in the liver tissue homogenates of experimental mice exposed to BPA, although RSV regulated the expression of these genes when compared with BPA treated experimental mice. CPT-I, CPT-II, and CACT genes are located in the mitochondria and are involved in the metabolism and transportation of carnitine. Hence, this study confirms that BPA exposure induced oxidative stress, upregulated gene expression, and impaired lipid and amino acid metabolism in experimental mice.

## 1. Introduction

Among all the environmental pollutants, the endocrine-disrupting effects of bisphenol A (BPA) are the most extensively studied and well established [1,2]. Its estrogenic activity was reported as early as the 1930s. It is used as a component of polycarbonate containers and plastics. BPA is found in the coating materials of jars and cans used for the food storage and dental materials [3]. The use of BPA as a color developing agent in the manufacture of thermal printing paper used for payment and cash receipts is another common source of exposure to human beings. Due to its omnipresent nature, BPA has been a subject of great research and has invoked the special interest of researchers due to its unavoidable contact with human beings that leads to the development of various diseases [1,4,5].

The exposure to BPA in our surroundings is inescapable. It is astonishing that BPA appears at quantifiable levels in the greater part of examined individuals. Diverse studies have measured the occurrence of BPA in urinary samples, but only a few of them have measured BPA levels in the blood. The amount of unconjugated BPA was approximately 1 ng/mL according to these studies [6].

With over six billion pounds currently produced per annum, BPA is among the most abundantly produced chemicals [1]. Many studies have demonstrated the detectable levels of BPA in the biofluids of supposedly unexposed human populations worldwide [7,8,9,10].

**Figure 1:**
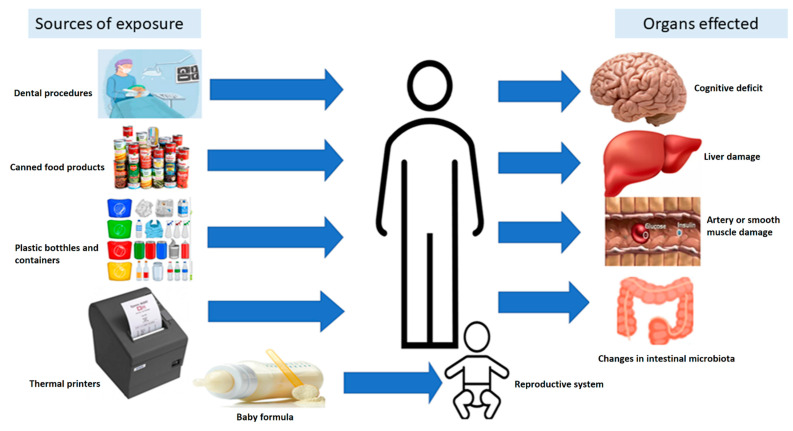
Schematic represention of the sources of BPA exposure and its impact on some of the organs in humans.

It has been found that 5 mg/kg/day of BPA did not exhibit adverse effects in experimental rats [3]. Based on the available information, about 50 μg/kg/day BPA levels in food and/or beverages may be consumed daily by any individual over a lifetime without any serious health considerations [11,12]. This dose exceeds the 95^th^ percentile level of BPA intake for adults, i.e., 1.5 μg/kg/day [12]. Different sources of BPA and the organs they affect in human beings are illustrated in Figure 1.

The use of omics studies, such as transcriptomics and proteomics, has found widespread applications in biological sciences as these techniques provide substantial information about the genotype; however, they have limited scope in phenotype studies. This limitation has led to increased interest in metabolomics, which is essentially a study of small metabolites that are close to the phenotype. In terms of studying the metabolic changes associated with exposure to environmental toxicants, metabolomics is among the most powerful tools [13,14].

This technique involves the high-throughput simultaneous screening and quantification of various small molecules, such as amino acids, carbohydrates, fats, or other products associated with cellular metabolic functions in biological tissues, cells, or fluids. Such extensive biochemical profiling provides information related to the genotype exposure to toxicants, diet, and microbiota. The relative ratios and levels of metabolites are reflective of metabolic function, and disturbances often indicate some problems. Therefore, quantitatively measuring metabolite concentrations has facilitated researchers in discovering new genetic sites responsible for the regulation of these small molecules, or their comparative ratios, in plasma, serum and other tissues [15,16,17]. MS-based techniques used to quantify metabolite pools are the most famous [17,18]. The use of metabolomics to interpret the effect of BPA can prove to be a promising technique as it involves a series of biochemical reactions, which results in small endogenous metabolites.

Metabolomics come at the end of the “omics” cascade (from genomics, transcriptomics, proteomics, to metabolomics) since the changes in the metabolome comes as the organism’s last retaliation in response to environmental exposure and/or contamination; thus, making metabolomics a highly useful technique for evaluating changes occurring at the cellular level in pathophysiological states [19]. Despite there being a clear link between oxidative stress and the metabolome, only limited literature is available about this interesting link.

Resveratrol (RSV) is available as a nutritional supplement due to its diverse health benefits. It is a polyphenolic stilbenoid, having two phenol rings that are linked to each other through an ethylene bridge. It has two isomeric forms cis and trans. The trans RSV (trans-3,5,4′-trihydroxystilbene) form is more prevalent. It has diverse health benefits, including a role in cancer treatment, via cellular responses such as cell cycle arrest, and cell apoptosis; it exhibits anti-proliferative effects in cancer cells. RSV has also been reported to act as a defense agent against oxidative stress [20,21,22,23]. The current study aimed to investigate the hazardous effects of BPA at the molecular level to understand its toxicity on the metabolism of biomolecules. Different parameters, such as oxidative stress and the detection of amino acids and lipids, along with the expression of carnitine palmitoyltransferase I (CPT-I), carnitine palmitoyltransferase II (CPT-II), lecithin–cholesterol acyltransferase (LCAT), carnitine O-octanoyltransferase (CROT), carnitine-acylcarnitine translocase (CACT), and 5-methyltetrahydrofolate-homocysteine methyltransferase (MTR) genes related to lipid homeostasis, were selected for analysis. The liver was chosen as the tissue to be tested as it is the major site of metabolism and plays a pivotal role in the metabolism of various biomolecules, as well as the immunomodulation, the synthesis of bile and numerous serum proteins, hormone catabolism, and the detoxification of xenobiotic toxicants. We also aimed to explore if the plant-based bioactive compound, i.e., RSV, has any therapeutic potential on BPA-induced metabolomic perturbations. Indeed, this study planned to determine the level of disruption present in lipid and amino acid metabolism, and the level of antioxidant enzymes due to BPA-induced toxicity in an experimental animal model, followed by the ameliorative effect of RSV against BPA toxicity through the investigation of biochemical profiling.

## 2. Materials and Methods

### 2.1. Preparation of BPA Solution

BPA is available in a small white pellet form. It was first dissolved in a small volume of ethanol and then in distilled water. The final volume of BPA solution was adjusted before its administration into the experimental animals.

### 2.2. Preparation of Resveratrol Solution

Resveratrol is available in the form of a coarse powder. For the administration of the RSV into the mice, it was blended in corn oil, and the concentration of RSV adjusted in the corn oil according to the body weight of the experimental animals.

### 2.3. Experimental Design

This study was conducted on 25 Wistar albino mice at nine weeks of age and weighing 30 ± 5 g. The mice were fed a pellet diet and had access to water *ad libitum*. The experimental protocols of this study followed the guidelines of the “Institutional Review Board (IRB) of Government College University Faisalabad (GCUF)” Faisalabad, Pakistan with the authorized reference number Ref. No. GCUF/ERC/37. The mice were randomly divided into five groups as given below:

Group 1: Control group

This group contained 5 mice that were given normal saline via the oral route.

Group 2: BPA group

This group had 5 mice that were exposed to a 50 mg/kg dose of BPA via oral gavage.

Group 3: BPA+RSV-CRN—group

This group had 5 mice that were first exposed to a 50 mg/kg dose of BPA and then treated with an 8 mg/kg dose of RSV blended in corn oil via oral gavage.

Group 4: RSV-CRN only group/reference group

This group had 5 mice that were treated with an 8 mg/kg dose of RSV blended in corn oil via oral gavage.

Group 5: Corn oil group

The mice (*n* = 5) in this group received corn oil alone.

### 2.4. Dosing Schedule

All treatments were given to the respective mice group daily via the oral gavage route for 4 weeks. During the study period, the body weight of the mice in all the groups was monitored regularly. After 4 weeks, the mice were fasted overnight. After anesthetizing the mice, whole blood was taken out from their body via cardiac puncture for serum and plasma separation. They were then sacrificed by dislocating the cervical bone and their liver was obtained after dissection. The following parameters were studied: oxidative stress, perturbations in amino acids, gene expression, and lipid metabolism.

### 2.5. Collection of Liver for Antioxidant Test and mRNA Expression of Genes

The liver was used for the evaluation of the mRNA expression of genes (Table 1), and antioxidant status. For the mRNA expression and antioxidant test, the liver was taken in zip-lock bags and kept in ice till further analysis.

### 2.6. Estimation of Biomarkers of Oxidative Stress and Lipid Peroxidation

Estimation of lipid peroxidation, i.e., malondialdehyde (MDA) and oxidative stress markers: glutathione peroxidase (GPx), superoxide dismutase (SOD,) glutathione (GSH), and catalase (CAT) were carried out by using their corresponding ELISA kits (Elabscience Biotechnology Inc., Houston, TX, USA) according to the manufacturer’s specifications.

### 2.7. Gene Expression

The effect of BPA on the expression of genes relating to lipid and amino acid metabolism was determined by using qRT-PCR. Approximately 100 mg of the frozen liver was cut with a sterile scissor and homogenized with 1 mL of TRIzol reagent (Biobasic BS410A-MA18DR0J, Markham, ON, Canada). A Thermo Scientific RevertAid First Strand cDNA Synthesis Kit (Thermo Fisher Scientific, Waltham, MA, USA) was used to obtain the cDNA according to the manufacturer’s specifications.

The primers for preselected genes (Table 1) were designed by the Custom DNA Oligos—Eurofins Genomics and were diluted as per the instructions given by the manufacturer. The prepared qRT-PCR plate was run on a qRT-PCR machine and at thermal cycles, 95 °C for 10 min, followed by 40 cycles (denaturation for 15 s at 95 °C, and annealing for 30 s at 60 °C) by using a real-time PCR machine. β-actin was used as an internal reference or housekeeping gene. The fold change of the genes was calculated using the Livak method. The sequence and size of the expected reference and target genes are shown in Table 1.

### 2.8. Sample Preparation for the Analysis of Amino Acids

The blood was taken in an EDTA tube and centrifuged at 2000 rpm for 20 min at 4 °C to obtain the plasma. While separating the plasma, the cellular buffy coat should be avoided. The separated plasma was stored at 4 °C for further treatment.

We mixed the separated plasma with a 3% 5-sulfosalicylic acid (3 g of 5-SSA in 100 mL of distilled water) solution in a ratio of 1:1. The sulfosalicylic acid led to the precipitation of proteins in the plasma. The contents of the tube were mixed immediately and then allowed to stand for 30 min at 4 °C. Then, it was centrifuged at 10,000× *g* at 4 °C for 5 min until a clear supernatant was obtained. If lipids are present in the sample, they will form a top layer on the clear supernatant, so the pipette tip should be below this while removing the supernatant. The collected supernatant was dried by liquid nitrogen gas. The amino acid analysis was performed using an amino acid analyzer (AAA) and all the other stocks and standard solutions were prepared in accordance with the internal protocols of the AAA.

### 2.9. Sample Preparation for MS/MS Analysis

To obtain the serum, the whole blood was centrifuged at 3500× *g* for 10 min at 4 °C. The serum was stored at −80 °C till further analysis. Before the metabolomics analysis, 600 μL of cold methanol was added to 200 μL of the serum and shaken vigorously. The mixture was stored for 10 min followed by centrifugation at 12,000× *g* for 10 min at 4 °C. The supernatant was then filtered through a 0.22 μM polytetrafluoroethylene polymer (PTFE) before injection into an ion trap mass spectrometer for Tandem mass spectrometry (MS/MS).

### 2.10. General Conditions for Sample Analysis by MS/MS

- **Instrument:** Linear Ion Trap Mass spectrometer model; LTQ XL (Thermo Electron Scientific, Waltham, MA, USA) equipped with electro spray ionization source [24].
- **Solvent:** Methanol.
- **Mode of injection:** Direct insertion method.
- **Flow rate:** 9.8 μL/min.
- **Mode of ionization:** Both negative and positive scan ion modes.
- **Capillary voltage:** 4.7 kV
- **Capillary temperature:** 278 °C
- **Sheath gas flow rate:** 17 units
- **Auxiliary gas flow rate:** 6 units
- **Scanning mass range:** 50–2000 *m/z*
- **Fragmentation (MS/MS):** Various peaks were selected for fragmentation, using collision-induced dissociation (CID) energy ranging from 20–30
- **Software:** Xcalibur 2.0.7

The qualitative analysis of serum metabolomes by MS/MS analysis was performed at the National Institute of Biotechnology and Genetic Engineering (NIBGE), Faisalabad, Pakistan.

### 2.11. Statistical Analysis

The results were estimated as mean ± SD. A one-way ANOVA was used to determine the significant difference between the groups when the value of probability was considered as (*p* < 0.05) using GraphPad Prism 5 (GraphPad Software Inc., La-Jolla, CA, USA). The graphical data were also represented as the mean and SD.

## 3. Results

### 3.1. Effect on Body Weight

**Figure 2:**
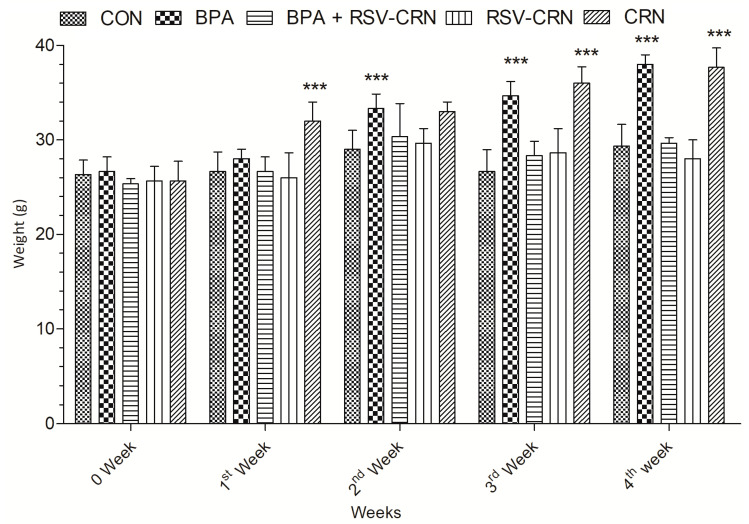
A graphical representation of weight gain in all groups over the experiment timeline. Week 0 represents the weight of the animals at the start of the study. Here, it can be seen in the 1st week bar charts that only the corn oil receiving group showed a significant increase in weight, which can be observed throughout the study. The BPA receiving group showed significant *p* < 0.05 weight gain in the 2nd week of the study as compared to week 0. This increase in weight of the BPA receiving group was also observed in the 3rd and 4th week of the study. At the end of the study, both the BPA receiving and corn oil receiving group showed significant *p* < 0.05 weight gain as compared to the start of the study and the control group. In the resveratrol receiving groups, i.e., BPA+RSV-CRN and RSV+CRN, the role of resveratrol in combating weight gain can be observed throughout the experiment timeline. Both groups showed restraint in weight gain as compared to the BPA and CRN group and showed a non-significant difference to the start of the study and among each other. The results were analyzed using a two-way ANOVA followed by Bonferroni’s post-test comparison. Here *** shows a highly significant difference, i.e., weight gain in the groups receiving corn oil and the group receiving BPA throughout the study as compared to week 0. Each group contained 5 animals.

The body weight of the mice in each group was recorded before the start of the study. After that, the body weight was recorded every week before eating for four weeks to track any changes in the weight of each group. It was observed that in the first week of the study, there was no significant weight gain in any group except the corn oil group, which showed an increase in their body weight. In the 2^nd^ week, a significant weight gain was observed in the group intoxicated with BPA as compared to that of the control group, which did not show any weight gain. The RSV receiving groups also showed a restraint in weight gain during the 2^nd^ week; however, their weight was significantly higher as compared to their weight at the start of the study period. At the end of the 3^rd^ week, the BPA receiving group showed a significant increase in their body weight as compared to the other groups. At the end of the study period, the weight gain in the BPA receiving group was significantly higher as compared to their body weight in previous weeks; although the RSV receiving groups, i.e., RSV-CRN and BPA+RSV-CRN, showed a restraint in weight gain as compared to the BPA and CRN groups. The CRN group showed a significant increase in weight throughout the experiment period. The restraint in weight gain in the RSV receiving groups hints at its ability to mitigate the effects of a high-fat diet and BPA induced weight gain. Consistent weight gain throughout the study in the BPA receiving group affirms its metabolism disrupting tendency. A graphical representation of the change of body weight of the mice in all the treated groups is shown in Figure 2.

### 3.2. Determination of Lipid Peroxidation and Oxidative Stress Biomarkers

**Figure 3:**
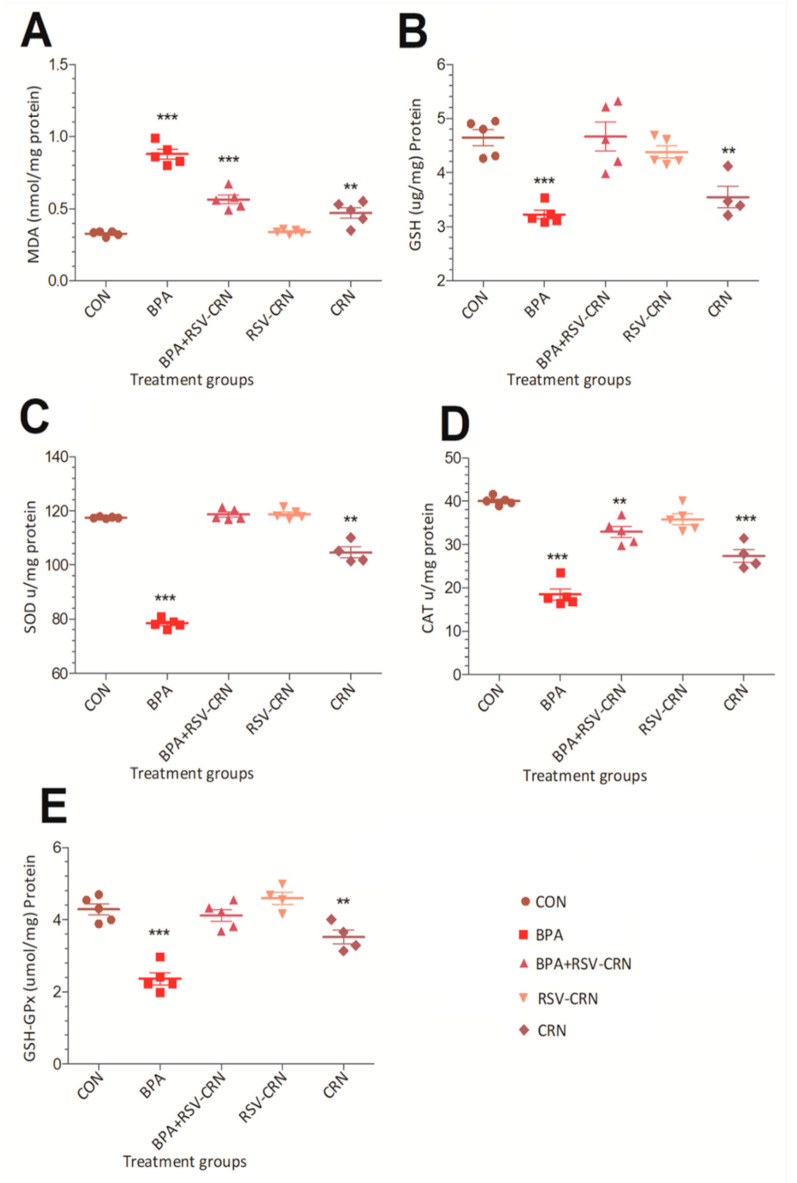
Effect of BPA on peroxidation biomarkers and antioxidant enzymes (**A**) MDA, (**B**) GSH, (**C**) SOD, (**D**) CAT, and (**E**) GPx, and the ameliorative effect of RSV. The level of GSH, SOD, CAT and GPx were decreased at the end of experiment while MDA levels were enhanced. The data were analyzed using one-way ANOVA. The results are expressed as the mean ± SD. ** shows the level of significance where *** means a highly significant difference from the control group. Abbreviations: GSH: Glutathione; SOD: Superoxide dismutase; CAT: Catalase; GPx: Glutathione peroxidase; MDA: Malondialdehyde; CON: Control group; BPA: BPA receiving group; BPA+RSV-CRN: BPA and resveratrol blended in corn oil group; RSV-CRN: resveratrol blended in corn oil group; CRN: Corn oil group.

The MDA level was markedly enhanced in the BPA treated group as compared to that of the control group, which showed a normal level of MDA at 0.32 mmol/mg (Figure 3A). After BPA exposure, the level soared to 0.83 mmol/mg, which showed a significant difference (*p* < 0.05) from the control group. RSV administration significantly lowered the MDA level to 0.06 mmol/mg, which is significantly lower (*p* < 0.05) than that in the BPA exposure group. Although the level of MDA was also somewhat higher (0.61 mmol/mg) in the corn oil treated group than in the control group, the MDA levels in the RSV treated group were comparable to the control group at 0.33 mmol/mg. This confirms the tendency of BPA to elevate the MDA level and also confirms that RSV is a potential therapeutic agent that has the tendency to ameliorate the deleterious effects of BPA.

The level of antioxidant enzymes, i.e., CAT, SOD, GSH, and GPx, were significantly lowered (*p* < 0.05) in the group treated with BPA (Figure 3B–E), while the group receiving RSV-CRN in addition to BPA showed a significant increase in the level of antioxidant enzymes as compared to that of the BPA group. The difference between the control and the corn oil group was also significant (*p* < 0.05), as the corn oil group also showed a certain decrease in the levels of said enzymes, while the difference between the RSV-CRN group and control group was statistically non-significant as shown in Figure 3B–E.

### 3.3. Effect of BPA on Gene Expression of Lipid Metabolism Related Genes

**Figure 4:**
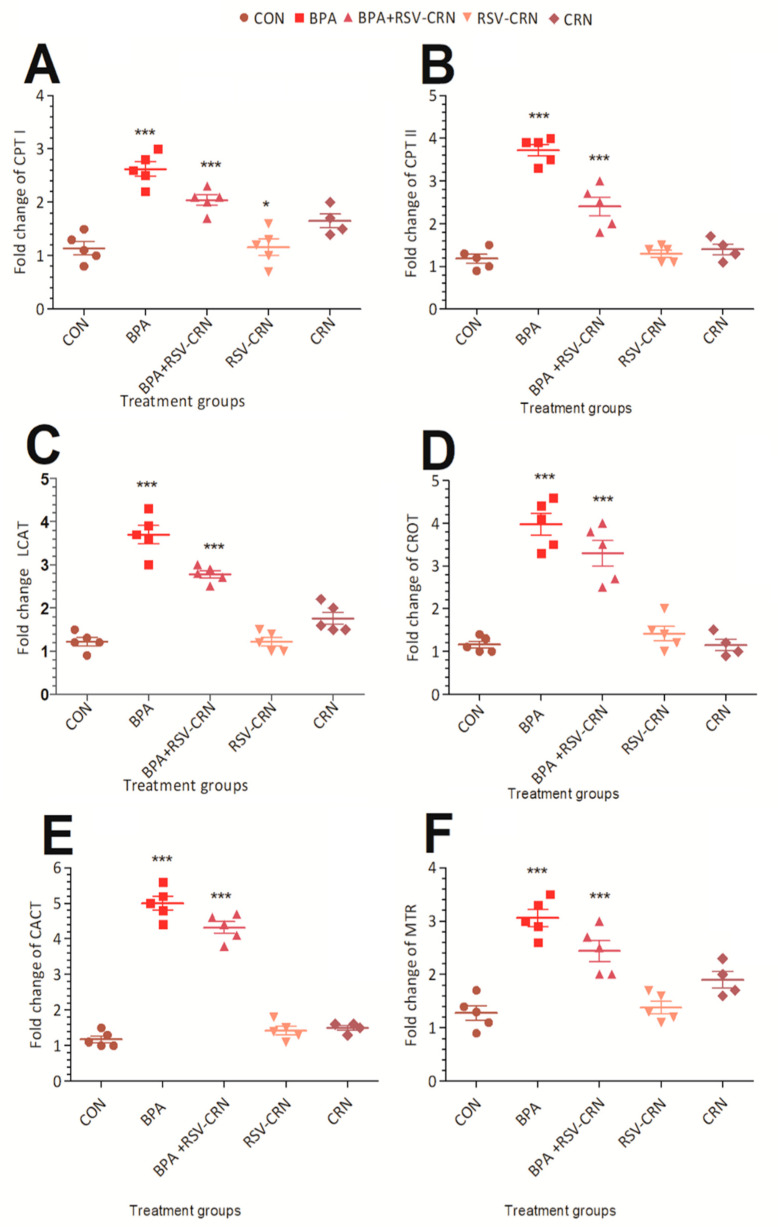
Graphical representation of the effects of BPA on mRNA expression of the (**A**) CPT I, (**B**) CPT II, (**C**) LCAT, (**D**) CROT, (**E**) CACT, and (**F**) MTR genes in all groups, i.e., CON, BPA, BPA+RSV-CRN, RSV-CRN, and CRN. The quantitative analysis was performed at the end of the experiment. Data were analyzed using one-way ANOVA. * is used to show the significance of the results where *** means a highly significant difference from the control group. Abbreviations: CPT I: Carnitine palmitoyltransferase I; CPT II: Carnitine palmitoyltransferase II; LCAT: Lecithin–cholesterol acyltransferase; CROT: Carnitine O-octanoyltransferase; CACT: Mitochondrial carnitine/acylcarnitine carrier protein; MTR: 5-methyltetrahydrofolate-homocysteine methyltransferase.

The mRNA expression of lipid metabolism related genes (Table 1) was studied in the liver tissue homogenate experimental mice. Carnitine palmitoyl transferase I (CPT-I) is an enzyme responsible for the conversion of long-chain acyl-CoA species to their corresponding long-chain acyl-carnitines to facilitate their transport inside the mitochondria. It is present in the outer mitochondrial membrane. Gene expression of CPT-I was upregulated markedly in the group receiving BPA as compared to the control group (Figure 4A). The RSV-CRN group did not show any significant upregulation, while the corn oil group showed an upregulation trend. No significant difference between the BPA treated group and the group receiving the co-treatment of RSV was found.

Carnitine palmitoyl transferase II (CPT-II) also showed a marked elevation in gene expression in the BPA receiving group. It is responsible for catalyzing the reconjugation of long and very-long-chain acylcarnitines to coenzyme A next to mitochondrial translocation. The co-treatment group receiving RSV in addition to BPA did not show any difference from the BPA receiving group (Figure 4B).

Lecithin–cholesterol acyltransferase (LCAT) is responsible for encoding the enzyme necessary for esterifying extracellular cholesterol; a step crucial in cholesterol transport. Our study showed a significant increase in the level of LCAT in BPA-exposed mice as compared to the control (Figure 4C).

Carnitine O-octanoyltransferase (CROT) is important in transporting medium- and long-chain acyl-CoA. It also trans-esterifies fatty acyl chains [13]. CROT is a member of the carnitine acyltransferase family, and is responsible for catalyzing the reversible conversion of L-carnitine + acyl-CoA ⇄ acyl-L-carnitine + CoASH. In HepG2 human liver cells, overexpression, and gene silencing of CROT decreases and increases long-chain fatty acids, respectively. There was a significant difference between the CON and BPA groups, i.e., gene upregulation. In addition, a significant (*p* < 0.05) decrease was seen in the levels of this gene in the group receiving RSV-CRN in addition to BPA, while the rest of the groups, i.e., CON, RSV-CRN, and CRN did not show any significant difference from each other (Figure 4D).

The mitochondrial carnitine-acylcarnitine translocase (CACT) carrier protein transports free carnitine out of mitochondria in exchange for carnitine-fatty acid complexes that are used for breakdown during tissue fasting states. It is essential for energy homeostasis. BPA showed an upregulation in gene expression while the results of the group receiving the BPA+RSV-CRN treatment showed the potential of resveratrol in lowering the level of gene expression (Figure 4E).

5-methyltetrahydrofolate-homocysteine methyltransferase (MTR) plays a role in making the enzyme methionine synthase, which is important in making the amino acid methionine from homocysteine. It plays an important role in methionine homeostasis. The levels of MTR in the liver tissue homogenate, as determined at the end of the 28 day period, showed a significant difference (*p* < 0.05) between the CON and BPA groups, i.e., gene upregulation. In addition, a significant (*p* < 0.05) decrease was seen in the levels of the gene in the group receiving RSV-CRN in addition to BPA; although the rest of the groups, i.e., CON, RSV-CRN, and CRN, did not show any significant difference from each other. LCAT is responsible for encoding the enzyme necessary for esterifying extracellular cholesterol. Our study showed a significant increase in the level of LCAT in the BPA-exposed mice as compared to the control (Figure 4F).

### 3.4. Detection and Quantification of Amino Acids

**Figure 5:**
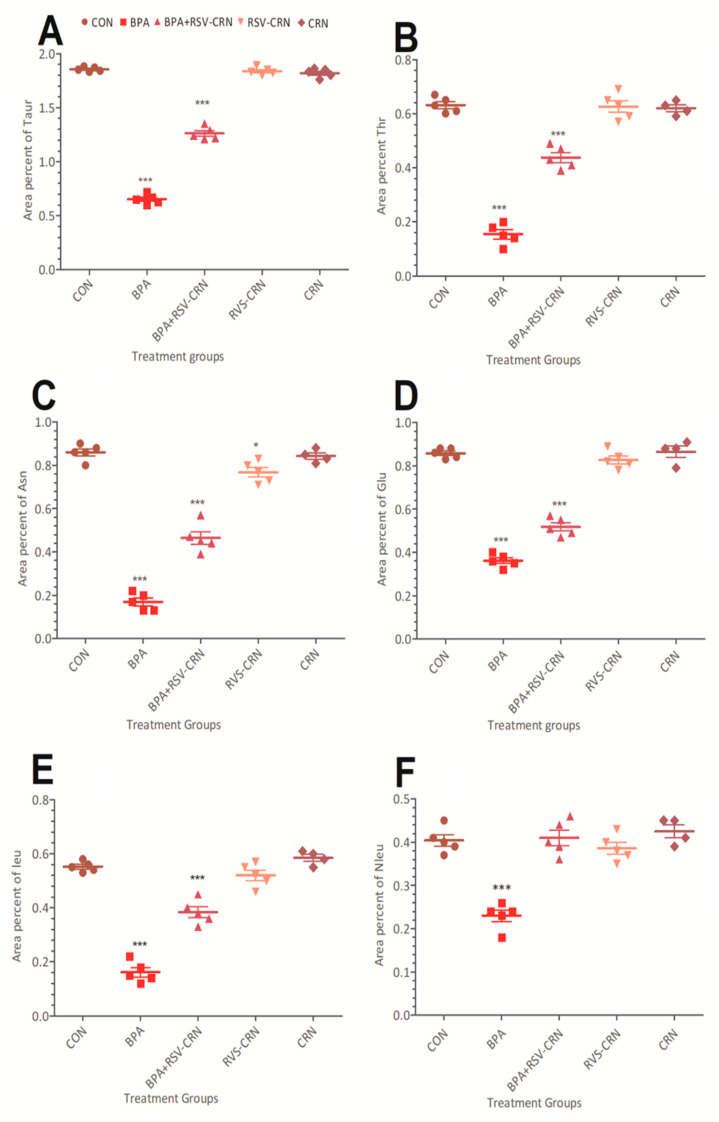
Graphical representation of the results of the amino acid analyzer: (**A**) taurine, (**B**) threonine, (**C**) asparagine, (**D**) glutamic acid, (**E**) leucine, and (**F**) norleucine in the plasma of different mice groups, i.e., CON, BPA, BPA+RSV-CRN, RSV-CRN, and CRN groups, respectively. The quantitative analysis was performed at the end of the experiment of 28 days using a one-way ANOVA followed by Tukey’s test to compare all pairs of columns. Each group contained 5 animals. The error bar represents the mean ± SD. * are used to illustrate the statistical significance of results where *** represents a highly significant difference in comparison to the control group while * shows a statistically low significance. Abbreviations: Taur: Taurine; Thr: Threonine; Asn: Asparagine; Leu: Leucine; Nleu: Norleucine; Glu: Glutamic acid; CON: Control group; BPA: BPA group; BPA+RSV-CRN: BPA and resveratrol blended in corn oil group; RSV-CRN: Resveratrol blended in corn oil group; CRN: Corn oil group; ANOVA: Analysis of variance.

Mice plasma was analyzed using AAA. The results included the screening and quantification of 16 amino acids, out of which six were chosen due to the significance of the results (Figure 5).

The group treated with BPA showed a significant decrease in the level of taurine, threonine, asparagine, leucine, and norleucine when compared to the control group, BPA+RSV-CRN group, and CRN group (Figure 5). When the group treated with BPA was compared with the one that received RSV-CRN followed by BPA, the result showed a significant (*p* < 0.05) improvement, i.e., a decrease in the area percent of taurine due to the ameliorative effect of the RSV. Glutamic acid was also significantly reduced in the BPA treated group.

### 3.5. MS/MS Analysis

Two groups, i.e., the BPA receiving and BPA+RSV-CRN, were selected for MS/MS analysis of the serum of the experimental mice. Out of all the compounds identified in the screening, four important lipid metabolites were identified from both the positive and negative modes of the full MS/MS scan. The compounds detected in both the BPA receiving and BPA+RSV-CRN group, their molecular formula, molecular weight, precursor *m*/*z*, and product ion *m*/*z* are elaborated in Table 2 and discussed below.

### 3.6. Sphinganine

**Figure 6:**
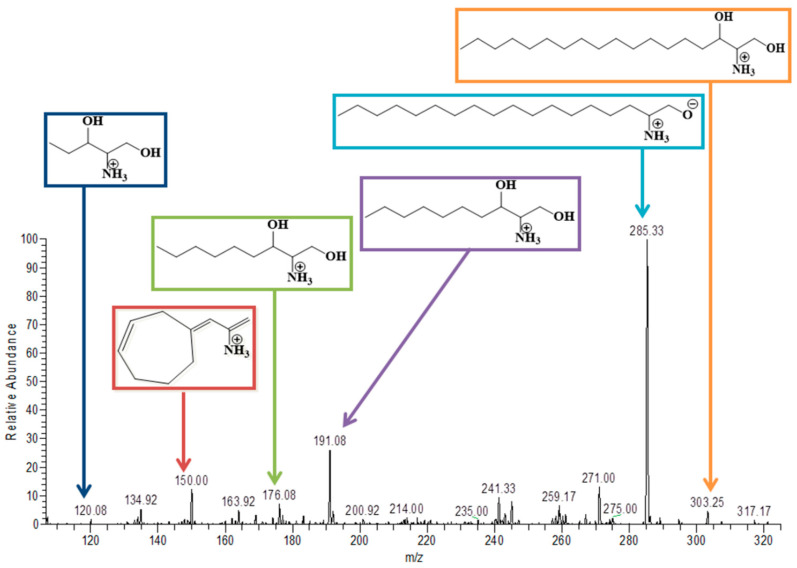
MS/MS spectrum of Sphinganine in positive ion mode.

This molecule was detected in the serum of both the BPA group and co-treatment group receiving RSV in addition to BPA. It was detected in the positive ion mode at 303.2. The MS spectrum also showed sphinganine fragments (Figure 6) after the removal of the alkyl chain of 8, 9, 13, and 14 carbons and showed its peak at *m*/*z* of 191, 176, 120.08, respectively. The peak at 190.3 overlapped with another peak at *m*/*z* of 191.08, which was thought to be due to the presence of an isotope. It also showed a fragment after the rearrangement at *m*/*z* of 150.

### 3.7. Phytosphingosine

**Figure 7:**
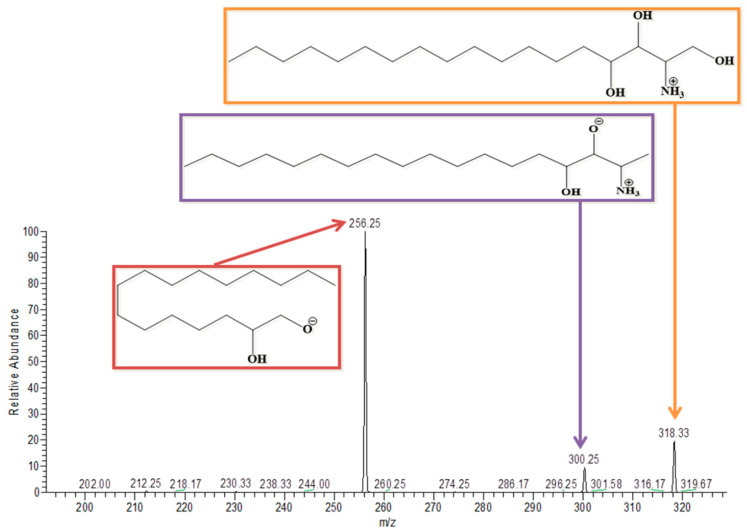
MS/MS spectrum of Phytosphingosine in positive ion mode.

Phytosphingosine has a molecular weight and was detected at *m*/*z* 318 in positive ion mode. Removal of a single water molecule gives a peak at 300 and the resultant 2-hydroxyethyl amine gives a peak at 256, both of which are highlighted in the spectrum in Figure 7.

### 3.8. Lysophosphatidylcholine

**Figure 8:**
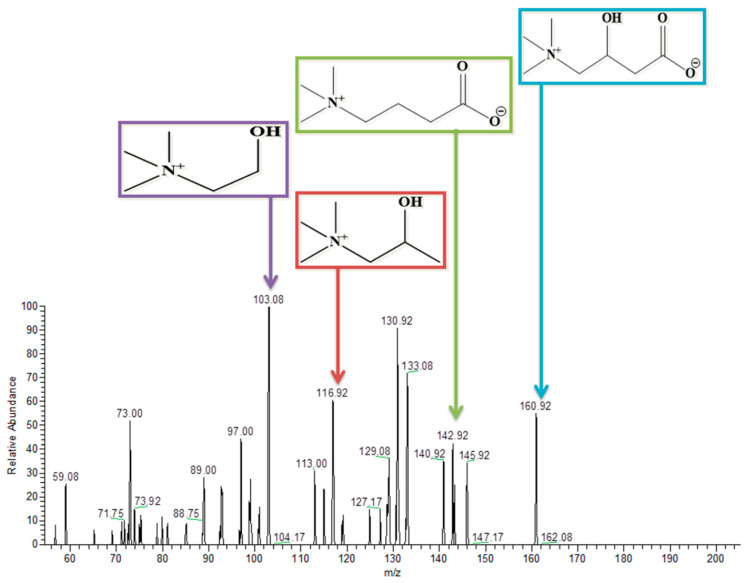
MS/MS spectrum of Lysophosphatidylcholine in positive ion mode.

**Figure 9:**
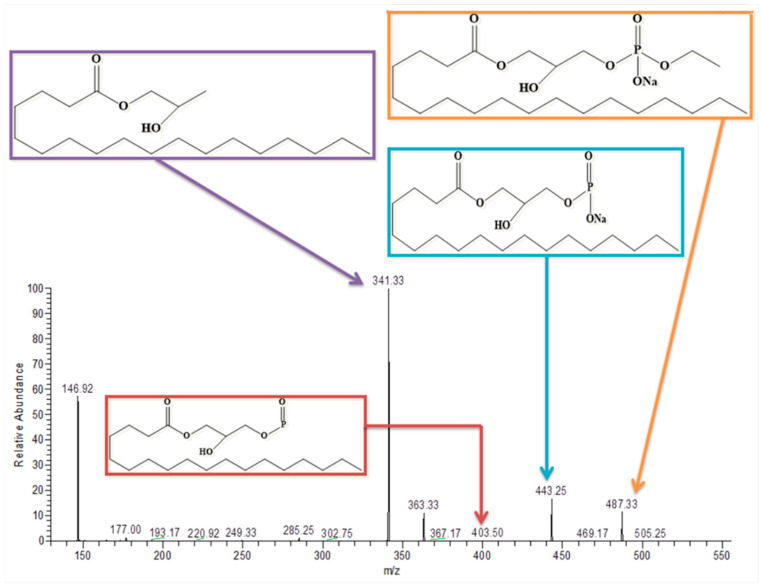
MS 2 of Lysophosphatidylcholine.

In positive ion mode, the peak present at 546 was identified as LysoPC. LysoPC has a molecular weight of 523; after the addition of a sodium ion, it showed its peak at *m*/*z* 546 as shown in Figure 8 and Figure 9, respectively.

### 3.9. L-Carnitine

**Figure 10:**
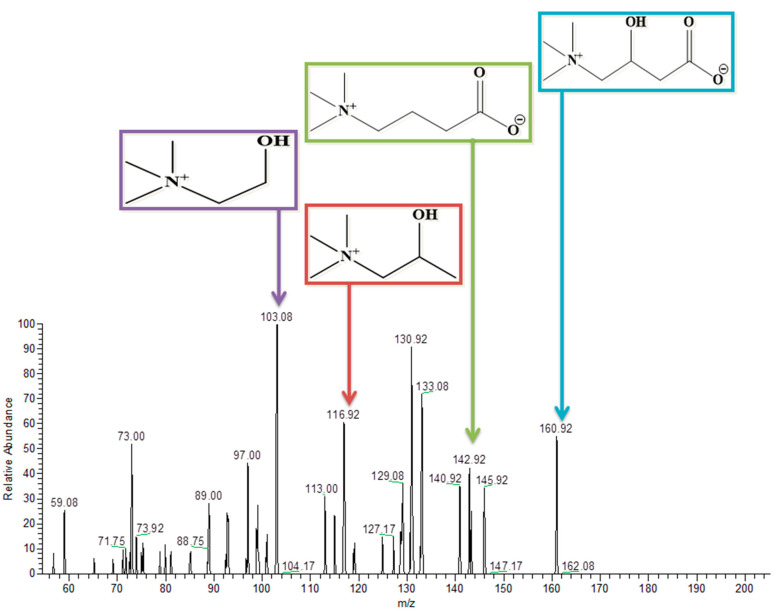
Structure and MS/MS spectrum of L-Carnitine in negative ion mode.

L-Carnitine has a molecular weight of 162. It was detected at 160.75 in the negative ion mode of the tandem mass spectrometry. Removal of a water molecule, carboxylic group, and ethanoic acid from the parent molecule gives peaks at 142.9, 117, and 103.08, respectively, as represented in Figure 10. Similarly, Table 3 also describes these results briefly.

## 4. Discussion

BPA has long been on the radar due to its widespread use and detrimental effects on health and the environment. It has been labeled an endocrine disrupter and many regulations have been put upon its usage in plastic wares. Although regulations are in place, one may not fully protect oneself from BPA exposure as many studies have reported the presence of BPA in supposedly unexposed populations. In 2020, a study conducted by [7] found that 75% of the study population had detectable levels of BPA in biological samples.

The current study found that MDA had increased in the BPA receiving group, which is consistent with the results of previous studies [25].

In this study, we also attempted to find the effect of BPA exposure on antioxidant enzymes; the results indicated that BPA induced oxidative damage even when the exposure was supposedly at a safe daily intake. Levels of antioxidant enzymes, namely SOD, GPx, and CAT, were elevated in liver tissues, which are the main sites for detoxifying and antioxidant activity. In one study, it was found that the levels of an antioxidant enzyme increased in a rat model at a dose of 50 mg/kg/day [26]. This is consistent with our results; thus, further establishing the evidence against BPA in disrupting the redox ratio of hepatic cells and causing oxidative stress as a result.

Disruption of the liver redox status leads to many destructive cascades in the hepatic environment; thus, restoration of antioxidant enzymes in hepatocytes is essential for normal hepatic activity. One of the most effective methods for restoring normal function against the damage caused by xenobiotics is the use of natural products that have the potential to heal the injury at a molecular level without causing any other damage. RSV was chosen as a natural compound in this study to investigate its effectiveness against BPA.

Similarly, it was also found that 10 mg/kg body weight of RSV reversed the oxidative stress in experimental rats with cholestasis [27]. The authors reported an elevation in the GSH level and a reduction in the MDA level in rats. Pandey et al. found a time and dose-dependent increase in the level of GSH in erythrocytes with the use of RSV [28]. A study conducted in 2020 tried to explain the antioxidant activity of RSV on oxidative stress induced by BPA exposure in the cortical collecting duct cells of mouse kidneys [29]. The results found that RSV was effective in diminishing the damage caused by oxidative stress in mouse kidney cortical duct cells.

Our findings indicate that RSV elevated the levels of SOD, GPx, CAT, and GSH in BPA induced liver damage and oxidative stress. These findings are useful in establishing the potential use of resveratrol for the reversal of oxidative stress caused by xenobiotics and environmental pollutants including BPA.

It has been found that BPA significantly increases the level of CPT-I in male *G. rarus*. This increase indicated that BPA may increase the tendency of β-oxidation in fatty acids while simultaneously increasing lipid synthesis. The authors also found that BPA increased triglyceride levels in the body [30].

The effect of BPA on lipid metabolism in marine *Gobiocypris rarus* was evaluated in a 28 day study. The authors found that BPA increased triglyceride levels in male fish, probably via the up-regulation of lipid synthesis. They also found an increased level of CPT-I, which indicates a higher tendency of β-oxidation in fatty acids with BPA exposure; however, the authors concluded that it might be reverted by increased lipogenesis [30].

**Scheme 1:**
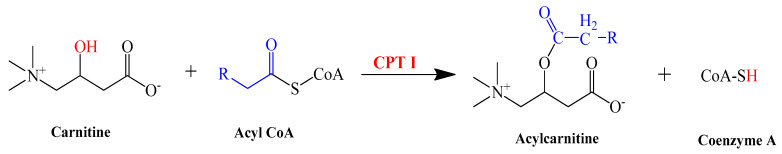
Synthesis of acylcarnitine from carnitine.

CPT-I is present at the outer mitochondrial membrane. CPT-I is responsible for catalyzing the reaction between carnitine and acyl-CoA (Palmitoyl-CoA and octadecenoyl-CoA) and leads to the formation of acylcarnitine (Palmitoyl carnitine and octadecenoyl carnitine) and CoA [31,32] as shown in the following reaction (Scheme 1). This reaction takes place in the intermembrane space of mitochondria.

**Scheme 2:**
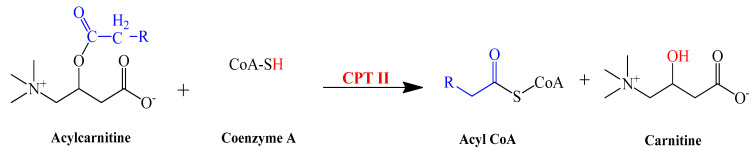
Synthesis of carnitine from acylcarnitine.

CPT-II is responsible for converting the acylcarnitine (Palmitoyl carnitine and octadecenylcarnitine) back into free carnitine and long-chain acyl-CoA as shown in the following reaction (Scheme 2) that takes place in the mitochondrial matrix before entering into the citric acid cycle [14,31,32].

CACT is located on the inner mitochondrial membrane. Its function is the translocation of free carnitine produced inside the mitochondrial matrix back into the cytoplasm for the next cycle reaction [31]. It also catalyzes the translocation of acylcarnitine produced in the presence of CPT-I in the intermembrane mitochondrial space to the inner mitochondrial matrix [32]

In our study, we also found an increased level of mRNA expression of selected genes related to β-oxidation of fatty acids, i.e., CPT-I and CPT-II in hepatocytes, but an increase in the body weight of BPA-exposed mice matches the trend of the previous study and might suggest that BPA increases lipid synthesis and the β-oxidation of the fats simultaneously. This indicates that BPA might have effects at sub-transcriptional levels.

The LCAT gene is responsible for making the LCAT enzyme, which plays an important role in the removal of cholesterol from blood, arteries, and other tissues, taking them to the liver for metabolism and removal from the body. The study showed that levels of LCAT were increased in the hepatic tissue homogenate in the BPA induced group and decreased in the group receiving resveratrol in addition to BPA. It may be explained as the body’s way of coping with increased circular levels of cholesterol and increased lipogenesis.

Shin et al. performed an H-NMR based metabolomic study on *Daphnia manga*, exposing it to BPA at a sublethal concentration, and found that levels of alanine, valine, isoleucine, leucine, arginine, phenylalanine, and tyrosine were increased in the species. They ascribed that these changes might be a result of glucose and lactate and may be due to a decrease in protein synthesis [14]; thus, in consequence, the level of amino acid was increased to counterbalance the toxic stress.

We implied that the quantitative measurement of amino acids in our study was via a non-targeted approach. The results showed a significant decrease in the concentration of taurine, which is responsible for heart and brain health. Its deficiency may lead to problems with different metabolic processes resulting in high blood pressure, kidney dysfunction, and thyroid hyperactivity. A decrease in taurine concentration was observed in a group receiving BPA, while no other group showed such a decrease. RSV showed efficacy in lowering the effect of BPA.

Elmetwally et al. attempted to gauge the effects that BPA might have on the proliferation, adhesion, and relocation of porcine trophectoderm cells [33], and the expression of the transporters of arginine, and amino acid synthesis. They observed that BPA had an inhibitory effect on the release or synthesis of the amino acids asparagine, threonine, taurine, tryptophan, and ornithine into the cell culture by pTr2 cells. They concluded that BPA had an adverse effect on the expression of transporters for cationic amino acids such as arginine.

**Figure 11:**
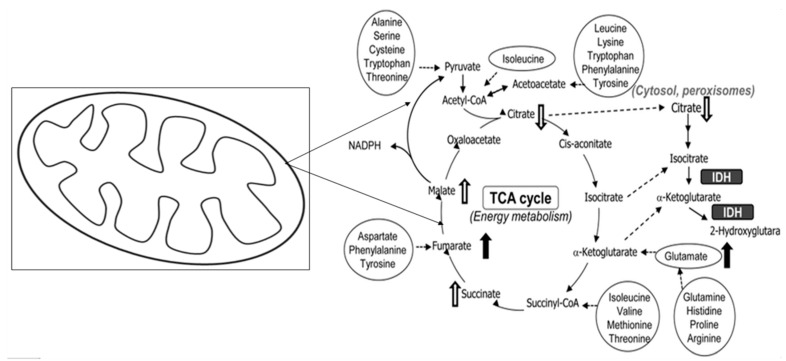
Schematic representation of the metabolomic pathway of amino acids taking place in mitochondria.

Our results showed a decrease in the levels of amino acids including asparagine, glutamic acid, leucine, norleucine, etc.; other amino acids were also detected but did not show any significant differences from the control, so only these six were included in the study results due to significant results. Figure 11 shows the metabolomic pathway of the amino acids that occurs inside the mitochondria.

There is strong evidence of altered lipid metabolism after BPA exposure as reported in previous studies [34]. For β-oxidation of long-chain fatty acids in mitochondria, they must first bind to carnitine. Carnitine facilitates the transportation of these long-chain fatty acids inside mitochondria. Ekman et al. performed metabolomic profiling of fish mucus merman and found a preeminent level of carnitine at 10 µg/L BPA exposure; thus suggesting that BPA has the potential to interfere in the β-oxidation of long-chain fatty acids [35].

## 5. Conclusions

This study was designed to test whether a sub-lethal dose of BPA possesses any threat to the metabolome and whether the natural plant-based bioactive compound, i.e., RSV will be effective in correcting those perturbations or not. The level of amino acids, including taurine, threonine, leucine, norleucine and glutamic acid were low, which suggested that BPA interferes with amino acid metabolomic pathways. Out of these amino acids, leucine appears to have a role in promoting β-oxidation of fatty acids; thus, promoting lipid metabolism. RSV showed encouraging results in this regard. Experimental mice simultaneously co-administered with RSV showed restored plasma levels of amino acids near to the control. In the case of biochemical profiling, the levels of SOD, CAT, and GPx in the liver tissue homogenates were also significantly lower (*p* ≤ 0.05), whereas the MDA levels were significantly high (*p* ≤ 0.05) in the BPA-treated groups when compared with the control group. However, in the RSV-treated group, the results were significantly improved. It seems that RSV scavenged the ROS and restored the oxidative balance to normal level. The results of qRT-PCR for CPT-I, CPT-II, CROT, LCAT, and CACT and the MS/MS serum analysis also showed a significant elevation (*p* ≤ 0.05) in the BPA intoxicated group in contrast to the control group. In the groups that were administered RSV, it was observed that the levels were significantly lowered in contrast to the intoxicated groups.

## 6. Future Perspectives

Metabolomics could prove to be a powerful tool in understanding the pathway and toxicity exerted by BPA at a molecular level. Further studies focusing on large populations and a longer period of exposure should also be conducted to explore a better understanding of the deleterious effects of endocrine disrupting chemicals via metabolomic tools using experimental animal models, human populations with long-term BPA exposure, and supposedly unexposed populations to confirm the exact pathway of toxicity in the impairment of metabolism.

## Figures and Tables

**Table 1:** List of the primers employed in the qRT-PCR analysis of the targeted genes.

Gene Name	Gene Symbol	Primer (5′–3′)		Target Size (bp)
Beta-actin	ẞ-Actin	Forward	CCCATCTATGAGGGTTACGC	150
		Reverse	TTTAATGTCACGCACGATTTC	
Carnitinepalmitoyltrans- ferase I	CPT-I	Forward	ATCCACCATTCCACTCTGCT	107
		Reverse	TGTGCCTGCTGTCCTTGATA	
Carnitinepalmitoyltrans-ferase II	CPT-II	Forward	CTGTCCACCAGCACTCTGAA	111
		Reverse	GCAACCTATCCAGTCATCGT	
Lecithin–cholesterol acyltransferase	LCAT	Forward	CTCCTTCTGGCTCCTCAATG	171
		Reverse	TCCTCTGTCTTTCGGTAGCAC	
Carnitine O-octanoyltransferase	CROT	Forward	AGACGGAAGGGAGATGGAG	168
		Reverse	AAGATGTGAAGGTAGATGCTGCT	
Mitochondrial carnitine/acylcarnitine carrier protein	CACT	Forward	TTCTCCACTGCTGCTCCTG	100
		Reverse	CCTGTCTGCTCCCATTCAG	
5-methyltetrahydrofolate-homocysteine methyltransferase	MTR	Forward	GGTTCGGTTGAAGAAGAGGA	112

**Table 2:** Lipids identified in the MS/MS spectra of mice serum.

Compound	M.F.	M.wt.	Polarity	Precursor Ion (*m*/*z*)	Product Ion (*m*/*z*)	BPA Group	BPA+RSV-CRN Group
Carnitine	C_7_H_16_NO_3_	162	Negative	160.75	142.9, 117 and 103.08	✓	✓
Sphinganine	C_18_H_39_NO_2_	302	Positive	303	285, 190.3, 176.3, 150, 119.92 and 106.	✓	✓
Phytosphingosine	C_18_H_39_NO_3_	317	Positive	318	300 and 256	✓	✓
Lysophosphatidylcholine	C_26_H_54_NO_7_P	523	Positive	546	487, 341, 404, and 443	✓	✓

**Table 3:** Comparison of results of different study groups.

Parameter	Bisphenol A	Bisphenol A + RSV	Corn Oil
Body weight	Increased	Decreased	Increased
Antioxidant enzymes	Decreased	Increased	Decreased
MDA level	Increased	Decreased	Increased
Amino acid levels	Decreased	Slightly Increased	Increased

## Data Availability

All data generated and/or analyzed during this study are included in this article.
